# Theoretical Investigation of the Hydrolytic Mechanism of α-Functionalized Alkoxysilanes as Effective Crosslinkers and the Difficulty of Deep Vulcanization in RTV Silicone Rubber

**DOI:** 10.3390/ma11091526

**Published:** 2018-08-24

**Authors:** Huihui Xu, Yanhong Gao, Zihou Liu, Yiling Bei

**Affiliations:** Key Laboratory of Special Functional Aggregated Materials, Ministry of Education, School of Chemistry and Chemical Engineering, Shandong University, Jinan 250100, China; 201411546@mail.sdu.edu.cn (H.X.); A2936755368@163.com (Y.G.); EBLX22@163.com (Z.L.)

**Keywords:** α-, γ-ethylenediaminemethyl trimethyl-ketoxime silane, hydrolytic activity, ab initio calculation, density functional theory, RTV silicone rubber

## Abstract

The reactions between α-, γ-ethylenediaminemethyl trimethyl-ketoxime silane (α-, γ-EAMOS) and H_2_O were investigated on the geometries of stationary points, the reaction pathway (IRC), thermodynamic and kinetic analysis by density functional theory (DFT) at the B3LYP/6-311G (d, p) level. Interestingly, the results showed that the hydrolysis activity of α-EAMOS is higher than that of γ-EAMOS, due to the influence of an amino substituent in position α-C on silicon. α-EAMOS can be used as a superior crosslinker for room temperature vulcanized (RTV) silicone rubber to achieve rapid crosslinking without a toxic catalyst. Besides, compared with the reaction between α-EAMOS and H_2_O, the reactivity between α-EAMOS and hydroxy siloxane (HO–Si(CH_3_)_2_–OSiH_3_) was discussed. Particularly, it revealed that the deep vulcanization of RTV silicone rubber is difficult.

## 1. Introduction

Carbofunctionalized trialkoxysilanes (X–(CH_2_)*_n_*–Si(OR)_3_, where X is functional amines, chlorine, carboxyl etc., *n* = 1, 2, 3 is called α-, β-, γ-functionalized alkoxysilane, respectively) play a great role in organosilicon chemistry. They are widely used as coupling agents to connect inorganic and organic materials possessing various structures and properties [1,2]. And they are also used as crosslinkers for room temperature vulcanized (RTV) silicone rubber. The OR groups of trialkoxysilanes react with H_2_O to form silanols, which then condense with the polydimethylsiloxane (PDMS) to produce an elastomer with three-dimensional network structure [3,4]. The corresponding RTV silicone rubbers with excellent properties are widely used in sealants, adhesives, coatings, and spray foams [5,6,7].

Commercial crosslinkers used on a large scale have often been γ-functionalized alkoxysilanes (*n* = 3) because they are considered more stable than α- or β-functionalized alkoxysilanes (*n* = 1 or 2, β-effect makes β-functionalized alkoxysilanes unstable) [8,9]. However, the influence of the functional group on Si–OR of γ-functionalized alkoxysilanes is much smaller than that of α-functionalized alkoxysilanes because the γ-group lies far away from Si. When it comes to crosslinkers for silicone rubber, it is usually necessary to add a toxic catalyst, such as organotin, to complete the rapid vulcanization at room temperature. 

Now, some α-silane-terminated prepolymers crosslink faster than the corresponding prepolymers produced from conventional γ-functionalized alkoxysilanes [10]. So, α-functionalized alkoxysilanes get more attention in silicon chemistry. For example, chloromethyltriethoxysilane was used as a self-catalytic crosslinker in water-based silicone rubber, which then had low modulus and high elongation [11]. The use of α-amine alkoxysilanes bearing piperazidine improved the stability of silicone rubber [12]. Meanwhile, various α-amine functionalized alkoxysilanes as crosslinkers accelerated the curing rate of the silicone rubber [13]. Just now, a series of α-amine ketoximesilanes [14] were prepared to be used as auto-catalyzed crosslinkers in RTV silicone rubber [15]. Thereby, it was simply proved by using density functional theory (DFT) calculations that α-amine ketoximesilanes have fast hydrolysis rates [16].

Although there are many studies on α-functionalized alkoxysilanes, the effect of the α-functional amino group is not fully understood yet. The geometries of α-, γ-functionalized silanes and their reaction processes with H_2_O need further investigation to find the hydrolytic mechanism, which is related to the vulcanization of RTV silicone rubber. Besides, as for the deep vulcanization of RTV silicone rubber, it is that the crosslinker first transforms to silanol (Si–OH). When there is rare water involved, the reactions between the crosslinker and H_2_O and between the crosslinker and HO–PDMS are competitive. Particularly, the deep vulcanizing process of RTV silicone rubber is difficult [17,18,19]. But, there are rare studies accounting for this.

In this regard, we studied the reactions between α-, γ-ethylenediaminemethyl trimethyl-ketoxime silane (EAMOS) and H_2_O, respectively. They were simulated by DFT calculations for the geometries of stationary points, and thermodynamic and kinetic analysis. To reveal the difficulty of deep vulcanization of RTV silicone rubber, the reaction between α-EAMOS and H_2_O and the reaction between α-EAMOS and HO–Si(CH_3_)_2_–OSiH_3_ were compared.

## 2. Computational Methods

Here, the computational methods referred to the default convergence limit of B3LYP/6-311G (d, p) of DFT in Gaussian 03 (C. 02, Jinan, China) [20]. DFT calculations were carried out using Becke’s three-parameter hybrid function (B3LYP) level using the 6-311G (d, p) basis set. The geometry optimization was applied for the ground states and these ground states were assumed to be a singlet state. The theoretical calculations were performed by this quantum set. All the above-mentioned calculations for geometry optimization were carried out with the Gaussian 03 quantum chemistry program-package.

**Figure 1:**
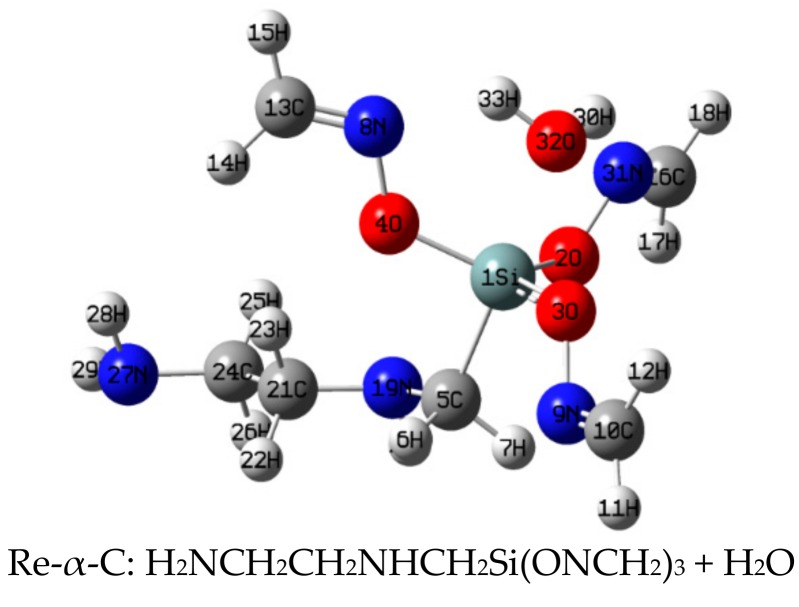

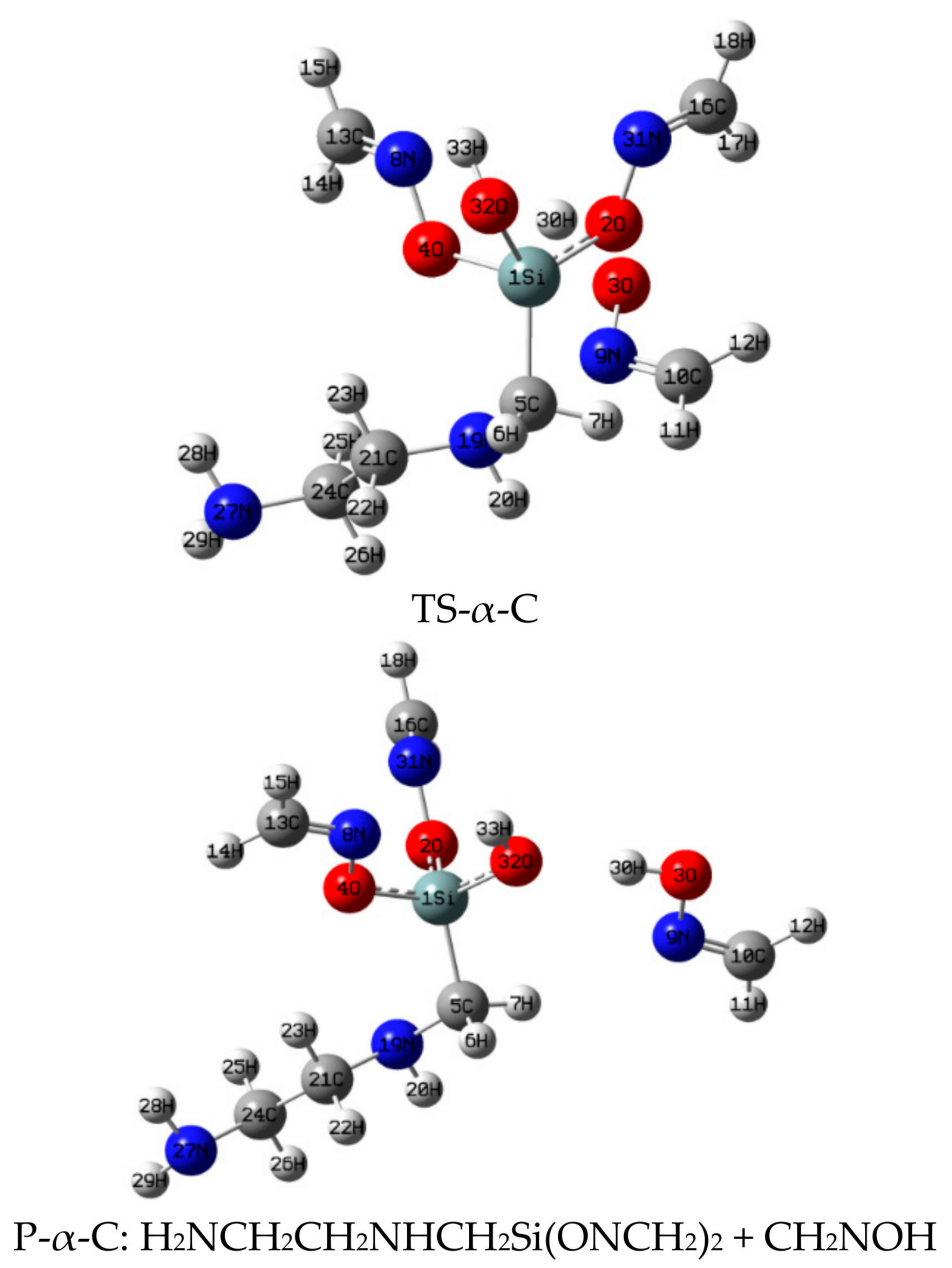
Geometries of stationary points of the reaction between α-ethylenediaminemethyl trimethyl-ketoxime silane (EAMOS) and H_2_O at B3LYP/6-311G** level.

The reactions between α-, γ-EAMOS and H_2_O were studied by using DFT at the B3LYP/6-311G (d, p) level. The molecules were optimized. The geometries of various stationary points and harmonic vibrational frequencies were calculated. Only one imaginary frequency for each transition state could be found. The reaction pathways were investigated and confirmed by intrinsic reaction coordinate (IRC) calculations (Figure 1). The changes of thermodynamic functions including entropy, enthalpy, free energies, and equilibrium constant were calculated through reactants, transition states, and products in Eyring transition state theory. Zero-point energy correction was also carried out at the B3LYP/6-311G (d, p) level. 

Having tried different reaction modes, the reaction path here needs the lowest energy. In order to guarantee the reality of the reaction path, the structures of transition states (the imaginary frequency is 1) were confirmed by vibration analysis. IRC of all the structures of transition states were calculated (Figure S1). The optimization was further performed along both sides of the IRC curve. One is reactants, and the other is products, which proved that the reaction path was reliable.

## 3. Results and Discussion

### 3.1. Stationary Points and Pathways with α-EAMOS and H_2_O as an Example

IRC calculation (Figure 1, Tables S1–S3) showed that in the reaction between α-EAMOS and H_2_O, the first step is the strong electronegative ^32^O (NBO charge: −0.918) of H_2_O, coordinated with two hydrogens (^30^H, ^33^H), moving to the electropositive ^1^Si (distance of ^1^Si–^32^O decreased from 2.038 Å to 1.803 Å, NBO charge: 2.156). Then, ^32^O and ^1^Si form a five-coordination intermediate by using the 3d orbital of Si. Afterwards, ^3^O (NBO charge: −0.732) leaves from ^1^Si (the ^3^O–^1^Si bond length increases from 1.710 Å to 2.065 Å), and ^30^H leaves from ^32^O (the ^30^H–^32^O bond length increases from 0.985 Å to 1.099 Å). This process crosses the barrier potential (78.8 kJ·mol^−1^), resulting in the transition state (TS-α-C). After that, ^32^O keeps close to ^1^Si, (the ^32^O–^1^Si bond length increases from 1.803 Å to 1.661 Å) resulting in an Si–O bond. ^30^H (NBO charge: 0.510) leaves from ^32^O and keeps close to ^3^O, (the ^30^H–^32^O bond length increases from 1.099 Å to1.814 Å and the ^30^H–^3^O bond length decreases from 1.368 Å to 0.975 Å). Finally, the product P-α-C is obtained. Here, TS-α-C was confirmed as first order saddle point by IRC analysis and the only imaginary frequency of transition is −841.9 HZ.

Besides, the reaction between γ-EAMOS and H_2_O follows a similar pathway shown in Figure S2 and Tables S4–S6.

### 3.2. The Highest Occupancy Orbit (HOMO) of Each Stationary Point of Reactions between α-, γ-EAMOS and H_2_O

**Figure 2:**
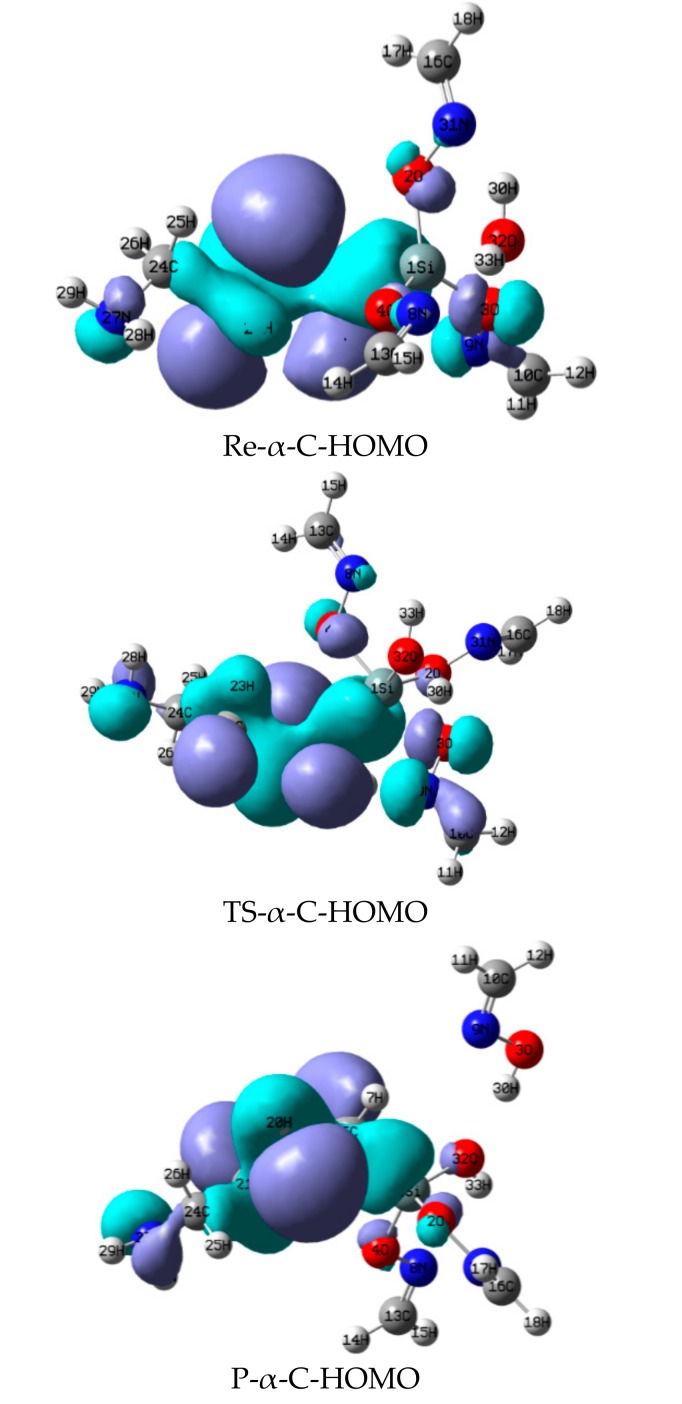
Highest occupied molecular orbital (HOMO) of stationary points of the reaction between α-EAMOS and H_2_O at the B3LYP/6-311G** level.

**Figure 3:**
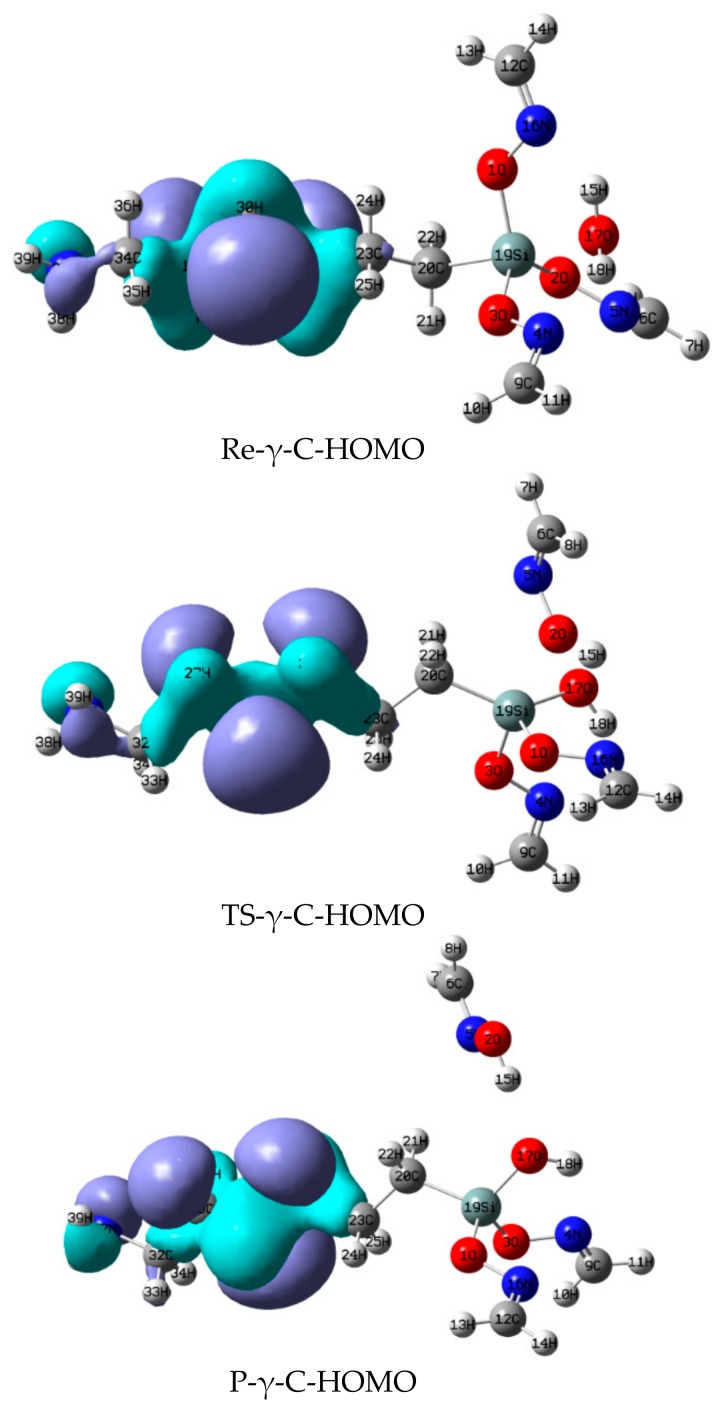
HOMO of stationary points of the reaction between γ-EAMOS and H_2_O at the B3LYP/6-311G** level.

The HOMO of the stationary points of the reactions between α-, γ-EAMOS and H_2_O in Figure 2 and Figure 3 were compared. In Re-α-C (H_2_N(CH_2_)_2_NHCH_2_Si(O–N=CEtMe)_3_), when H in the α position is replaced by an electronegative ethylenediamine group, ‘p’ electrons in N enter the 3d orbital of Si, resulting in p–π conjugation. Thus, the electronegativity of Si increases; it is easier to be attacked by nucleophiles. While electronegative OH in H_2_O comes close to Si to form a complex, the Si–O bond will break easily and separate. 

However, in Re-γ-C (H_2_N(CH_2_)_2_NH–CH_2_CH_2_CH_2_Si(O–N=CEtMe)_3_), the electron cloud of N will not conjugate with Si because the group lies far away from the Si atom. Thus, there is rare electron interaction on Si. As a result, the reaction between α-EAMOS and H_2_O is easier than the reaction between γ-EAMOS and H_2_O.

### 3.3. The Reaction between α-EAMOS and HO–Si(CH_3_)_2_–OSiH_3_

**Figure 4:**
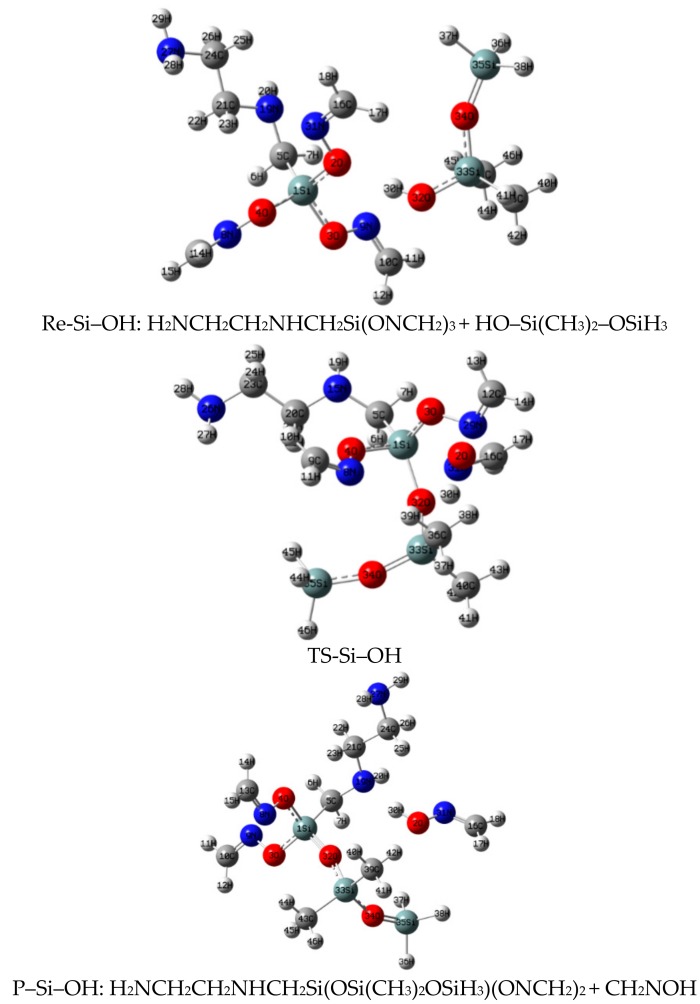
Geometries of stationary points of the reaction between α-EAMOS and HO–Si(CH_3_)_2_–OSiH_3_ at the B3LYP/6-311G** level.

IRC calculation (Figure 4, Tables S7–S9) showed that ^32^O in HO–Si(CH_3_)_2_–OSiH_3_ comes close to ^1^Si (distance of ^1^Si–^32^O from 4.127 Å to 1.827 Å). Then, ^32^O and ^1^Si share a five-coordination bond by using the 3d orbital of Si. Afterwards, ^2^O leaves from ^1^Si, (the ^1^Si–^2^O bond length increases from 1.684 Å to 2.035 Å), and ^30^H leaves from ^32^O, (the ^30^H–^32^O bond length increases from 0.969 Å to 1.076 Å). This process crosses the barrier potential, 118.9 kJ·mol^–1^, resulting the transition state (TS-Si–OH). After that, ^32^O keeps close to ^1^Si (distance of ^32^O–^1^Si decreases from 1.827 Å to 1.611 Å), resulting in a Si–O bond. ^30^H leaves from ^32^O and keeps close to ^2^O (the ^30^H–^32^O bond length increases from 1.076 Å to 3.004 Å and the ^30^H–^2^O bond decreases from 1.387 Å to 0.995 Å). Finally, the product P–Si–OH is obtained. Here, TS-Si–OH was confirmed as the first order saddle point by IRC analysis and the only imaginary frequency of transition is −468.9 HZ.

### 3.4. Thermodynamic and Kinetic Parameters

The entropy changes from reactant to transition state were calculated using vibration frequency. They were based on the equilibrium geometries of the transition states (TS-α, TS-γ-C, TS-Si–OH) and the products (P-α, P-γ-C, P–Si–OH). Then, the reaction rates were obtained by transition state theory, while entropy changes, enthalpy changes, Gibbs’ free-energy changes, and the equilibrium constants were calculated by using statistical thermodynamic method. Kinetic analysis further proved the possibility and practicality of these reactions. For calculating rate constants, some insertion points were selected in both sides of the transition states along the steepest descent energy curve calculated by IRC. Then, the Hessian matrices of these points, reactants, products, and the transition states were calculated.

Rate constant: k (T) = g(k_b_T/h) exp (ΔS^#^/R − ΔH^#^/RT)

g: Wigner correction factor, g = 1 + (hν^#^/k_b_T)^2^/24;
k_b_: Boltzmann constant (1.3806505(24) × 10^−23^ J·K^−1^);
T: thermodynamic temperature (K);
h: Planck constant (6.6260693(11) × 10^−34^ J·s);
R: molar gas constant (8.31441 ± 0.00026 J·mol^−1^·K^−1^);
V^#^: imaginary frequency of transition (cm^3^·mol^−1^);
ΔS^#^: standard molar activation entropy (J·mol^−1^·K^−1^);
ΔH^#^: standard molar activation enthalpy (kJ·mol^−1^).

Equilibrium constant: K (T) = exp (−ΔG × 1000/ (8.314T))

ΔG: Gibbs free energy (kJ·mol^−1^).

As shown in the kinetic calculations in Table 1 and Table 2, the rate constants will increase and the equilibrium constants will decrease gradually as the temperature increases. Interestingly, the reaction between α-EAMOS and H_2_O has high rate constants and equilibrium constants. In detail, the reaction between α-EAMOS and H_2_O has the highest rate constant which is three times that of γ-EAMOS and H_2_O, and ten orders of magnitude higher than that of α-EAMOS and HO–Si(CH_3_)_2_–OSiH_3_ at 298 K. This difference will decrease gradually as the temperature increases. 

As the thermodynamic calculations in Table S10 show, the reaction between α-EAMOS and H_2_O is entropy-increasing, exothermic, and spontaneous. The reaction between α-EAMOS and HO–Si(CH_3_)_2_–OSiH_3_ is exothermic, spontaneous, but entropy-decreasing. This is due to the polyreaction of two reactants from disorder to order. Besides, the reactions between α-EAMOS and H_2_O exhibit similar aviation energy (78.8 kJ·mol^−1^, Tables S2 and S5), while that of α-EAMOS and HO–Si(CH_3_)_2_–OSiH_3_ (118.9 kJ·mol^−1^, Table S8) is higher. 

In conclusion, α-functionalized silanes have a fast hydrolysis rate. Using α-functionalized ketoxime silanes as effective autocatalytic crosslinkers in RTV silicone rubber by decreasing the tack-free time and improving thermal stability (Figure S3) [15] or mechanical properties has been reported [16,21,22]. Besides, α-functionalized silanes are easier to react with H_2_O on the surface of silicone rubber but difficult to react with HO-PDMS (silicone rubber gum) in the vulcanizing system. It proved that the corresponding silicone rubber is difficult to deep vulcanize at room temperature. 

## 4. Conclusions

According to DFT calculations, the amine group on α-EAMOS is only one C atom away from Si, and the lone pair of electrons on the amine can be conjugated into the 3d orbital of Si, thus increasing the electronegativity of Si. Then, the hydrolyzable group on Si was attacked easily by H_2_O. But, there is no conjugation effect in γ-EAMOS, whose hydrolysis rate was lower than that of α-EAMOS. Interestingly, it proved that α-amino substituted alkoxy silanes could be used as efficient crosslinkers in RTV silicone rubber.

Besides, the reaction between α-EAMOS and H_2_O had a faster reaction rate, lower activation energy, and higher reaction trend than those of the reaction between α-EAMOS and HO–Si(CH_3_)_2_–OSiH_3_. So, α-functionalized ketoxime silanes could react with H_2_O quickly on the surface of silicone rubber but they are difficult to react with HO-PDMS. Then, there is rare cross-linking effect inside. As a result, the deep vulcanization of the corresponding silicone rubber is difficult. 

Here, DFT calculations were explanative and markedly consistent with our experimental results. Not only is this study important for basic research, it may also facilitate an increase in the use of α-functionalized ketoxime silanes to improve the properties of RTV silicone rubber in the silicon industry. 

## Figures and Tables

**Table 1:** Rate Constants k (T) calculated at the B3LYP/6-311G (d, p) level.

Rate Constants k(T) (s^−1^)			
T/K	α-C	γ-C	Si–OH
298	3.23 × 10^−1^	1.09 × 10^−1^	4.80 × 10^−11^
400	1.48 × 10^3^	3.66 × 10^2^	8.81 × 10^−6^
500	2.10 × 10^5^	4.12 × 10^4^	1.04 × 10^−2^
600	5.79 × 10^6^	9.45 × 10^5^	1.16 × 10^0^
700	6.29 × 10^7^	8.76 × 10^6^	3.35 × 10^1^
800	3.79 × 10^8^	4.61 × 10^7^	4.19 × 10^2^
900	1.54 × 10^9^	1.66 × 10^8^	2.99 × 10^3^
1000	4.78 × 10^9^	4.63 × 10^8^	1.45 × 10^4^
1100	1.21 × 10^10^	1.06 × 10^9^	5.26 × 10^4^
1200	2.63 × 10^10^	2.12 × 10^9^	1.55 × 10^5^
1300	5.09 × 10^10^	3.78 × 10^9^	3.86 × 10^5^
1400	8.98 × 10^10^	6.19 × 10^9^	8.45 × 10^5^
1500	1.47 × 10^11^	9.46 × 10^9^	1.67 × 10^6^

**Table 2:** Equilibrium Constants K (T) calculated at the B3LYP/6-311G (d, p) level.

Equilibrium Constants K(T)			
T/K	α-C	γ-C	Si–OH
298	1.04 × 10^14^	5.79 × 10^13^	9.28 × 10^11^
400	1.71 × 10^11^	1.06 × 10^11^	4.56 × 10^8^
500	4.12 × 10^9^	2.73 × 10^9^	5.10 × 10^6^
600	3.48 × 10^8^	2.41 × 10^8^	2.49 × 10^5^
700	6.01 × 10^7^	4.26 × 10^7^	2.84 × 10^4^
800	1.61 × 10^7^	1.17 × 10^7^	5.50 × 10^3^
900	5.80 × 10^6^	4.26 × 10^6^	1.53 × 10^3^
1000	2.56 × 10^6^	1.91 × 10^6^	5.45 × 10^2^
1100	1.32 × 10^6^	9.87 × 10^5^	2.34 × 10^2^
1200	7.55 × 10^5^	5.71 × 10^5^	1.15 × 10^2^
1300	4.71 × 10^5^	3.59× 10^5^	6.34 × 10^1^
1400	3.15 × 10^5^	2.41 × 10^5^	3.79 × 10^1^
1500	2.22 × 10^5^	1.71 × 10^5^	2.43 × 10^1^

## Supplementary Materials

The following are available online at http://www.mdpi.com/1996-1944/11/9/1526/s1, Table S1: B3LYP/6-311G**structural parameters of the reaction between α-EAMOS and H_2_O [bond length: Å; bond angle: (°); dihedral: (°)], Table S2: Absolute energies, zero-point energies and relative energies of reactants, transition states and products of the reaction between α-EAMOS and H_2_O calculated at B3LYP/6-311G** level, Table S3: The natural atomic charges of the reaction between α-EAMOS and H_2_O calculated at B3LYP/6-311G** level, Table S4: B3LYP/6-311G** structural parameters of the reaction between γ-EAMOS and H_2_O [bond length: Å; bond angle: (°); dihedral: (°)], Table S5: Absolute energies, zero-point energies and relative energies of reactants, transition states and products of the reaction between γ-EAMOS and H_2_O, calculated at B3LYP/6-311G** level, Table S6: The natural atomic charges of the reaction between γ-EAMOS and H_2_O, calculated at B3LYP/6-311G** level, Table S7: B3LYP/6-311G** structural parameters of the reaction between α-EAMOS and HO–Si(CH3)2–OSiH3 [bond length:Å; bond angle: (°); dihedral: (°)], Table S8: Absolute energies, zero-point energies and relative energies of reactants, transition states and products of the reaction between α-EAMOS and HO–Si(CH_3_)_2_–OSiH_3_, calculated at B3LYP/6-311G** level, Table S9: The natural atomic charges of the reaction between α-EAMOS and HO–Si(CH3)2–OSiH3, calculated at B3LYP/6-311G** level, Table S10: Entropy changes, enthalpy changes and Gibbs free energies calculated at B3LYP/6-311G (d, p) level, Figure S1: Total energy along IRC of reaction between α-EAMOS and H2O, Figure S2:B3LYP/6-311G** geometries of stationary points of the reaction between γ-EAMOS and H2O, Figure S3: TGA curves of the elastomer including α-silanes or MOS and PDMS (α-silanes: PDMS = 1:10).
