# Supplementary material for: Theoretical Investigation of the Hydrolytic Mechanism of α-Functionalized Alkoxysilanes as Effective Crosslinkers and the Difficulty of Deep Vulcanization in RTV Silicone Rubber
Source: Materials (Basel). 2018 Aug 24;11(9):1526. doi: 10.3390/ma11091526 (PMC6163510; doi:10.3390/ma11091526)
Supplement: Supplementary file 1 [file materials-11-01526-s001.pdf]

Theoretical Investigation of the Hydrolytic Mechanism of α-Functionalized Alkoxysilanes as Effective Crosslinkers and the Difficulty of Deep Vulcanization in RTV Silicone Rubber

Huihui Xu [1], Yanhong Gao [1], Zihou Liu [1] and Yiling Bei [1, *]

1 Key Laboratory of Special Functional Aggregated Materials, Ministry of Education; School of Chemistry and Chemical Engineering, Shandong University, Jinan 250100, P R China.

* Corresponding author: email: beiyiling@sdu.edu.cn; Tel:+86-0531-88362866

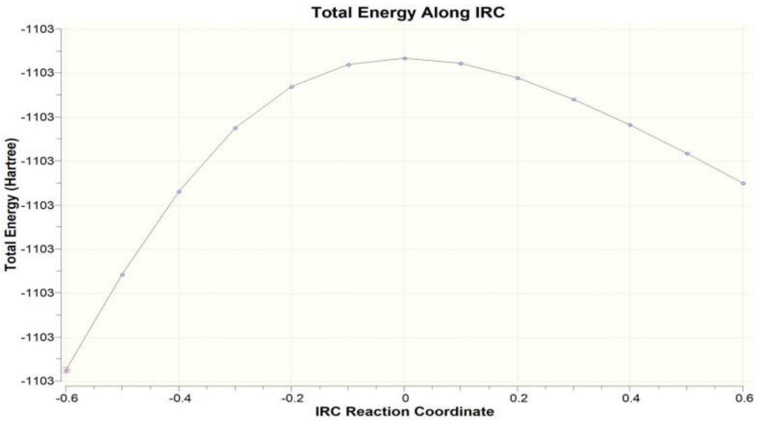

Figure S1 Total energy along IRC of reaction between α-EAMOS and H_2O.

IRC of all the structures of transition states were calculated. The optimization was further performed along both sides of the curve of IRC. One is reacts, and the other is products, which proved that the reaction between α-EAMOS and H_2O path was reliable.

Table S1. B3LYP/6-311G**structural parameters of the reaction between α-EAMOS and H_2O [bond length:Å;bond angle:(°);dihedral:(°)].

Species	^{1}Si-^{3}O	^{1}Si-^{32}O	^{32}O-^{30}H	^{30}H-^{3}O	^{1}Si^{32}O^{30}H	^{32}O^{1}Si^{3}O	^{33}H^{32}O^{1}Si^{3}O
Re-α-C	1.710	2.038	0.985	3.064	105.0	79.6	137.4
TS-α-C	2.065	1.803	1.099	1.368	87.3	70.3	137.4
P-α-C	3.917	1.661	1.814	0.975	121.5	37.3	170.8

Table S2. Absolute energies, zero-point energies and relative energies of reactants, transition states and products of the reaction between α-EAMOS and H_2O calculated at B3LYP/6-311G** level.

Species	E_{ZPVE}/ (kJ mol^{-1})	B3LYP/6-311G**		
		E/a.u.	E_T/a.u.	E_R/(kJ•mol^{-1})
Re-α-C	687.0930	-1103.1765	-1102.9148	0.0
TS-α-C	673.7236	-1103.1414	-1102.8848	78.7650
P-α-C	683.3756	-1103.2003	-1102.9400	-66.1626

Table S3. The natural atomic charges of the reaction between α-EAMOS and H_2O calculated at B3LYP/6-311G** level.

Species	^{1}Si	^{3}O	^{32}O	^{30}H	^{33}H
Re-α-C	2.156	-0.732	-0.918	0.510	0.509
TS-α-C	2.173	-0.714	-0.971	0.527	0.540
P-α-C	2.230	-0.560	-1.101	0.495	0.518

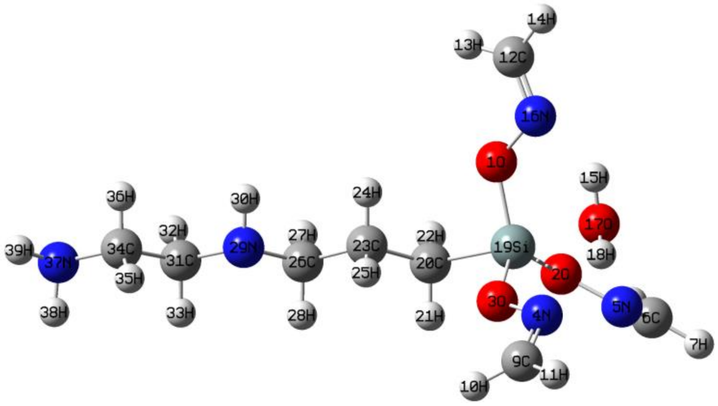

Re-γ-C: $H_2NCH_2CH_2NHCH_2CH_2CH_2Si(ONCH_2)_3+H_2O$

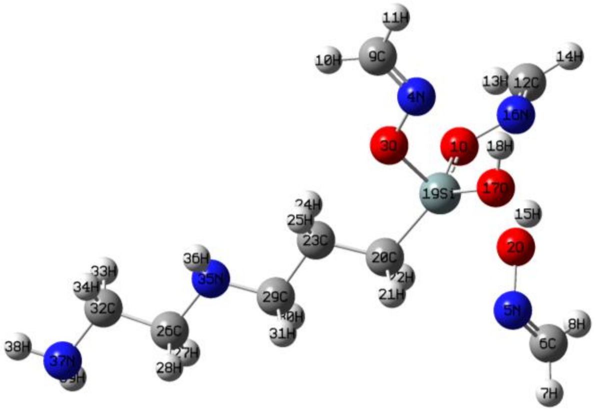

TS-γ-C

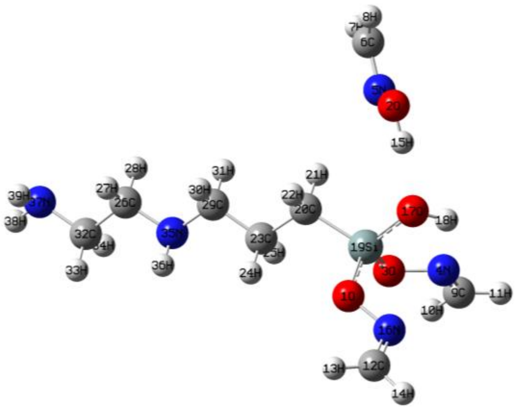

P-γ-C: $H_2NCH_2CH_2NHCH_2CH_2CH_2Si(OH)(ONCH_2)_2+CH_2NOH$

Figure S2 B3LYP/6-311G** geometries of stationary points of the reaction between γ-EAMOS and H_2O

Table S4. B3LYP/6-311G** structural parameters of the reaction between γ-EAMOS and H_2O [bond length:Å; bond angle:(°);dihedral:(°)].

Species	^{19}Si-^{2}O	^{19}Si-^{17}O	^{17}O-^{15}H	^{19}Si^{2}O^{17}H	^{2}O^{19}Si^{1}O	^{1}O^{19}Si^{2}O^{17}O
Re-γ-C	1.697	2.038	0.984	51.9	119.1	82.1
TS-γ-C	2.049	1.801	1.106	49.6	88.5	113.2
P-γ-C	3.864	1.659	1.817	22.2	125.8	80.7

Table S5. Absolute energies, zero-point energies and relative energies of reactants, transition states and products of the reaction between γ-EAMOS and H_2O, calculated at B3LYP/6-311G** level

Species	E_{ZPVE}/ (kJ mol^{-1})	B3LYP/6-311G**		
		E/a.u.	E_T/a.u.	E_R/(kJ•mol^{-1})
Re-γ-C	835.8181	-1181.8318	-1181.5135	0.0
TS-γ-C	821.8615	-1181.7967	-1181.4837	78.2399
P-γ-C	832.7312	-1181.8554	-1181.5382	-64.8499

Table S6. The natural atomic charges of the reaction between γ-EAMOS and H_2O, calculated at B3LYP/6-311G** level

Species	^{19}Si	^{2}O	^{17}O	^{15}H	^{18}H
Re-γ-C	2.182	-0.745	-0.918	0.510	0.515
TS-γ-C	2.197	-0.710	-0.974	0.527	0.540
P-γ-C	2.246	-0.565	-1.093	0.493	0.518

Table S7. B3LYP/6-311G** structural parameters of the reaction between α-EAMOS and HO-Si$(CH_3)_2$-$OSiH_3$. [bond length:Å;bond angle:(°);dihedral:(°)]

Species	^{1}Si-^{2}O	^{1}Si-^{32}O	^{2}O-^{30}H	^{32}O-^{30}H	^{1}Si^{32}O^{30}H	^{32}O^{30}H^{2}O	^{1}Si^{32}O^{30}H^{2}O
Re-Si-OH	1.684	4.127	1.944	0.969	14.1	172.1	168.8
TS-Si-OH	2.035	1.827	1.387	1.076	85.3	132.7	-3.4
P-Si-OH	4.178	1.611	0.995	3.004	89.8	108.2	-173.0

Table S8. Absolute energies, zero-point energies and relative energies of reactants, transition states and products of the reaction between α-EAMOS and HO-Si(CH_3)$_2$-OSiH_3, calculated at B3LYP/6-311G** level

Species	E_{ZPVE}/ (kJ mol^{-1})	B3LYP/6-311G**		
		E/a.u.	E_T/a.u.	E_R/(kJ•mol^{-1})
Re-Si-OH	929.8864	-1838.6866	-1838.3324	0.0
TS-Si-OH	924.0312	-1838.6390	-1838.2871	118.9352
P-Si-OH	936.0227	-1838.7165	-1838.3600	-72.4638

Table S9. The natural atomic charges of the reaction between α-EAMOS and HO-Si(CH_3)$_2$-OSiH_3, calculated at B3LYP/6-311G** level

Species	^{1}Si	^{2}O	^{33}Si	^{32}O	^{30}H
Re-Si-OH	2.263	-0.783	2.065	-1.083	0.511
TS-Si-OH	2.197	-0.715	2.127	-1.137	0.535
P-Si-OH	2.277	-0.596	2.122	-1.264	0.496

Table S10. Entropy changes, enthalpy changes and Gibbs free energies calculated at B3LYP/6-311G (d, p) level

	α-P-Re			γ-P-Re			Si-OH-P-Re		
T(k)	△H	△S	△G	△H	△S	△G	△H	△S	△G
298	-61.389441	57.437952	-78.5155775	-62.528908	58.504872	-79.9701045	-47.0988445	-17.526776	-41.8740995
400	-60.9824885	58.626208	-84.430829	-62.0589435	59.87304	-86.0087545	-47.3718965	-18.305	-40.049377
500	-60.7146875	59.228704	-90.327702	-61.736007	60.596872	-92.034277	-47.673829	-18.978624	-38.1826465
600	-60.5204005	59.584344	-96.2692085	-61.4970865	61.032008	-98.1175605	-47.9705105	-19.526728	-36.258155
700	-60.381249	59.801912	-102.2395955	-61.321178	61.308152	-104.237601	-48.2488135	-19.953496	-34.2811535
800	-60.28148	59.931616	-108.2257355	-61.195154	61.479696	-110.37602	-48.5008615	-20.288216	-32.2700205
900	-60.218468	60.002744	-114.225003	-61.105887	61.580112	-116.530192	-48.724029	-20.547624	-30.224756
1000	-60.1843365	60.0404	-120.226896	-61.048126	61.642872	-122.6922405	-48.913065	-20.75264	-28.161113
1100	-60.1738345	60.052952	-126.2314145	-61.01662	61.67216	-128.8569145	-49.081097	-20.911632	-26.0790915
1200	-60.1790855	60.048768	-132.2385585	-61.006118	61.680528	-135.024214	-49.222874	-21.032968	-23.981317
1300	-60.197464	60.032032	-138.243077	-61.011369	61.680528	-141.1915135	-49.343647	-21.133384	-21.8730405
1400	-60.2263445	60.011112	-144.24497	-61.0244965	61.667976	-147.3614385	-49.448667	-21.21288	-19.754262
1500	-60.2631015	59.986008	-150.2442375	-61.048126	61.655424	-153.5261125	-49.5405595	-21.271456	-17.6302325

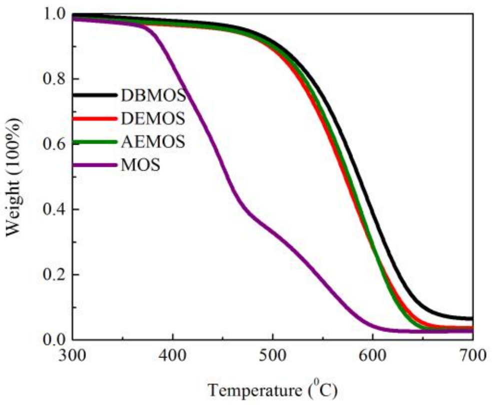

Figure S3. TGA curves of the elastomer including α-silanes or MOS and PDMS (α-silanes: PDMS= 1:10)

As shown in the Figure S3, the degradation temperature (the thermal weight loss consisted 5% of original weight) of elastomer with methyltris(methylethylketoxime)silane (MOS) was clearly lower than α-(N,N-di-n-butyl) aminomethyl tri(methylethylketoxime)silane (DBMOS), α-(N,N-diethyl) aminomethyl tri(methylethylketoxime)silane (DEMOS), α-(β-aminomethyl)aminomethyltri(methylethylketoxime)silane (AEMOS) by about 70 ℃. It illustrated the thermal stability of elastomer with α-silanes was better than methyltris(methylethylketoxime)silane (commercial crosslinker of silicone rubber).
